# Trends in cigarette smoking among adolescents and adults in South Korea

**DOI:** 10.4178/epih/e2014023

**Published:** 2014-10-28

**Authors:** Sunhye Choi, Yoonjung Kim, Suyeon Park, Jihye Lee, Kyungwon Oh

**Affiliations:** Division of Health and Nutrition Survey, Korea Centers for Disease Control and Prevention, Cheongju, Korea

**Keywords:** Cigarette smoking, Adults, Adolescents, South Korea

## Abstract

This report is to examine changes in the smoking prevalence among adults and adolescents and provide basic data for national health policies. Korea National Health and Nutrition Examination Survey from 1998 to 2013 and Korea Youth Risk Behavior Web-based Survey from 2005 to 2013 were used to estimate national adults and adolescents smoking prevalence. In 2013, current cigarette smoking prevalence among male adults and female adults was 42.1% and 6.2%, decreasing by 1.6% points and 1.7% points, respectively compared to 2012. Among adolescents, current cigarette smoking prevalence was 14.4% for male and 4.6% for female students, decreasing by 1.9%points and 1.3%points, respectively compared to 2012. The highest current cigarette smoking prevalence was observed among adults of lower household income or lower education level and among middle and high school students of lower perceived household economic status or lower perceived academic records. Current cigarette smoking prevalence among male adults has decreased since 2011, whereas among female adults, there were no statistically significant annual changes. Among middle and high school students, the prevalence for male students decreased since 2011 and for female students decreased since 2006. But the smoking prevalence did not meet the Health Plan 2020 target.

## INTRODUCTION

Smoking is a risk factor for various noncommunicable diseases (NCDs) such as cancer and cardiovascular and respiratory diseases [1], and in particular, smoking that begins during adolescence correlates closely with a direct negative impact on both physical and mental developments as well as with deviant and unhealthy behaviors [2]. Tobacco smoke contains more than 4,000 chemicals, at least 250 hazardous substances, and approximately 50 carcinogens; exposure to secondhand smoke increases the risks of premature death and occurrence of diseases in children and non-smokers [3,4]. Worldwide, 6 million smoking-related deaths are reported annually, and by 2030, the death toll is expected to increase to more than 8 million per year. Smoking-related diseases have been reported to account for half of all deaths in smokers; among smoking-related deaths, 10% are due to secondhand smoke [4].

In September 2011, at a United Nations (UN) high-level meeting on NCDs, world leaders agreed on a roadmap of concrete commitments to address the global burden of NCDs, including a commitment to establish multisectoral action plans and policies for the prevention and control of NCDs. To accelerate national efforts to address NCDs, in 2013 the World Health Assembly adopted a comprehensive global monitoring framework with 25 indicators and 9 voluntary global targets for 2025. Countries need to make progress on all these targets to attain the overarching target of a 25% reduction of premature mortality from the 4 major NCDs by 2025. Smoking target is a 30% relative reduction in prevalence of current tobacco use in persons aged 15 years and older. For the past 30 years, the World Health Organization (WHO) through its Ottawa and Bangkok Charters has made smoking a major issue and presented health promotion initiatives with the full support of related programs. In addition, South Korea also established targets for reducing smoking prevalence among adults and adolescents through its Health Plan 2020 (HP2020) [5]. Korea Centers for Disease Control and Prevention (KCDC) has continually monitored smoking trends among adults and adolescents through the Korea National Health and Nutrition Examination Survey (KNHANES) and Korea Youth Risk Behavior Web-based Survey (KYRBWS).

This report will examine trends in smoking prevalence among Korean adults and adolescents from KNHANES and KYRBWS data.

## MATERIALS AND METHODS

KCDC analyzed KNHANES from 1998 to 2013 and KYRBWS from 2005 to 2013 data to describe cigarette smoking prevalence among adults and adolescents.

KNHANES is a national surveillance system, which includes approximately 10,000 individuals aged 1 year and older from 3,840 households in 192 primary sampling units. The subjects of smoking in health behaviors were aged 12 years and older (this paper presented results of 19 years and older). The smoking behavior was surveyed by self-administration. The annual survey response rate was approximately 75%. KYRBWS data collected annually from approximately 75,000 students on grades 7-12 from 800 sampled schools through anonymous self-administered web-based survey. Annual response rate was 95% and over. The results from KNHANES were weighted to be representative of the Korean population, and the trend results from KNHANES were presented as age-standardized values based on 2005 population projection.

Current cigarette smokers were defined as adults aged 19 years and older who reportedly have smoked at least 5 packs of cigarettes (100 cigarettes) in their lifetime and are currently smoking everyday or someday; middle and high school students who reportedly have smoked for more than 1 day during the past 30days.

## RESULTS

**Figure 1.**
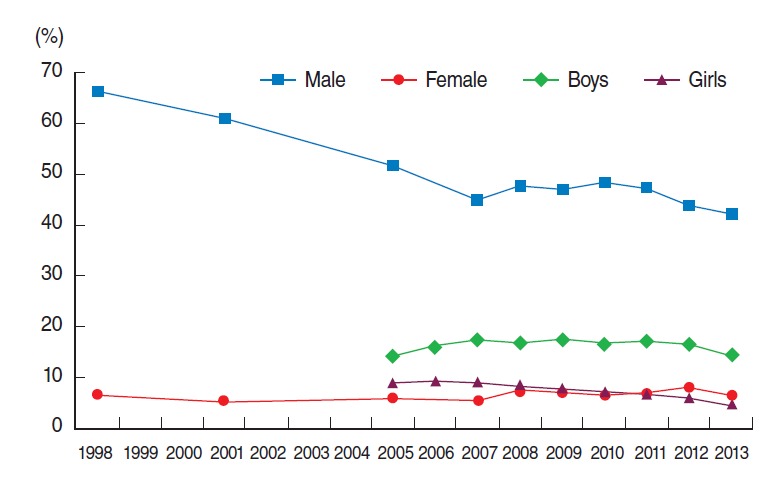
Trends in prevalence of current cigarette smoking^1,2^ in the Korea National Health and Nutrition Examination Survey during 1998 to 2013, and Korea Youth Risk Behavior Web-based Survey during 2005 to 2013. Proportions of adults were calculated using the direct standardization method and was based on a 2005 population projection. ^1^Adults (aged 19 years and older except for 1998 [20 years and older]; age-adjusted): percentage of adults who had smoked at least 5 packs of cigarettes (100 cigarettes) during their lifetime and who are currently smokers. ^2^Adolescents (aged 13–18 years except for 2005 [13–17 years]): percentage of students who smoked more than 1 day during the past 30 days.

During 2008-2013, the prevalence of current cigarette smoking (age-standardized) among male adults (≥19 years) did not change from 2008 (47.7%) to 2011 (47.3%) and declined from 2012 (43.7%) to 2013 (42.1%). For female adults, there were no significant differences between years, although the result of 6.2% in 2013 indicated a decrease of 1.7% points compared to 2012 (Figure 1). The prevalence of current cigarette smoking among male adults was higher than the HP2020 target of 29.0% (difference 13.1% points); among female adults, the prevalence of current cigarette smoking was very close to the HP2020 target of 6.0% [5]. Korean males are more than twice as likely to smoke than US male (20.5%) whereas Korean females are only half as likely to smoke as US females (15.3%) [6].

In 2013, the prevalence of current cigarette smoking was higher among males (14.4%) than among females (4.6%) in middle and high school students; differences from 2012 were 1.9% points and 1.3% points, respectively (Figure 1). The prevalence of current cigarette smoking among male students in 2013 was higher than the HP2020 target of 12.0%; however, among female students, prevalence was lower than the HP2020 target of 6.0% [5]. Korean male high school students were slightly higher than US male students (16.4%), whereas Korean female students were approximately 2.5 times lower than US female students (15.0%) [7].

**Figure 2.**
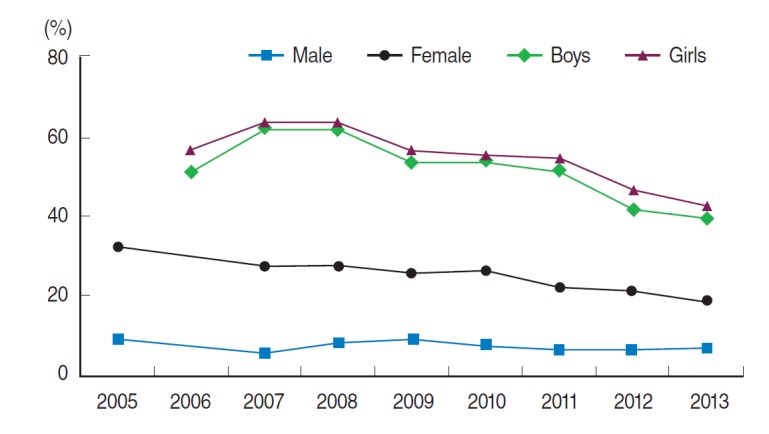
Trends in prevalence of secondhand smoke exposure at home^1,2^ in the Korea National Health and Nutrition Examination Survey during 1998 to 2013, and Korea Youth Risk Behavior Web-based Survey during 2006 to 2013. Proportions of adults were calculated using the direct standardization method and was based on a 2005 population projection. ^1^Adults (aged 19 years and older; age-standardized): percentage of current non-smokers who were exposed to cigarette smoke at home (2013: during the past 7 days). ^2^Adolescents (aged 13-18 years): percentage of students who were exposed to secondhand smoke at home on more than 1 day during the past 7 days.

The prevalence of secondhand smoke exposure at home (age-standardized) was higher among female adults (14.1%) than male adults (5.5%) in 2013. Females tend to be more exposed than males in the secondhand smoke at home (Figure 2). In addition, among adolescents (grades 7–12), the prevalence of secondhand smoke exposure at home was higher among female students (32.0%) than male students (29.5%); this prevalence continually declined since 2008 (46.0% males, 47.6% females). But the prevalence among adolescents was 2 times higher than among adults (Figure 2). The HP2020 targets of secondhand smoke exposure at home are 1.0%, 5.0%, and 5.0% for male adults, female adults and adolescents, respectively, meaning the current levels were higher than the targets [5].

**Figure 3.**
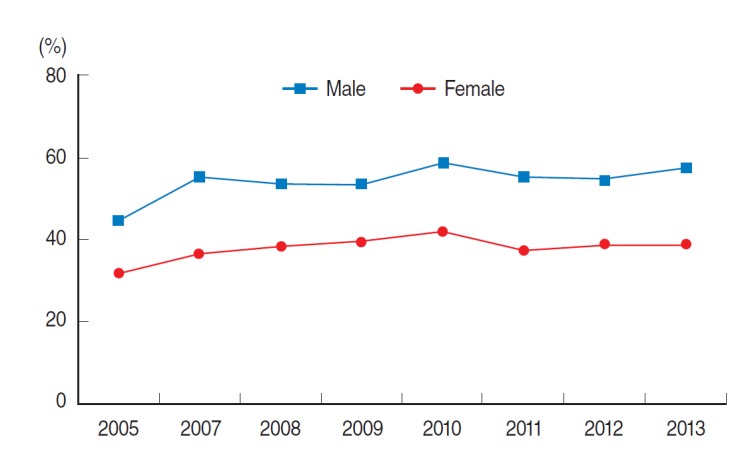
Trends in prevalence of secondhand smoke exposure in workplace^1^ in the Korea National Health and Nutrition Examination Survey during 2005 to 2013. Proportions were calculated using the direct standardization method and was based on a 2005 population projection. ^1^Adults (aged 19 years and older; age-standardized): percentage of current non-smokers who were exposed to cigarette smoke in workplace (2013: during the past 7 days).

The prevalence of secondhand smoke exposure in workplace (age-standardized) was higher among male adults (57.2%) than among female adults (38.7%) in 2013, indicating that the prevalence of secondhand smoke exposure in workplace for non-smokers remained high (Figure 3).

In 2013, the highest current cigarette smoking prevalence was observed among males aged 30-39 years old (54.5%), among females aged 19-29 years old (9.1%), among lower household income group by sex, or among lower education level group by sex (sex-adjusted and age-adjusted). In the lowest household income level, the prevalence for males and females was 46.1% and 8.9%, respectively and in the highest, the prevalence was 34.3% and 3.5%, respectively, representing differences of 11.8% points (males) and 5.4% points (females) between the highest and lowest levels. These results were higher than the HP2020 targets of 8.0% (males) and 1.5% (females) with respect to differences between household incomes [5]. Those with college and higher education level had the lowest prevalence of 40.2% among males and 2.1% among females. By occupations (aged 19–64 years old; sex-adjusted and age-adjusted), prevalence among males was the highest among technicians and mechanics (51.6%), followed by service and sales workers (51.4%) and simple laborers (49.3%); prevalence among females was the highest among service and sales workers (10.8%), followed by simple laborers (8.2%) and agriculture, forestry, and fishing workers (5.0%) (Table 1).

In 2013, the highest current cigarette smoking prevalence by school grades was among male students in grade 12 (22.8%) and female students in grade 11 (7.0%). Among middle and high school students, prevalence of current cigarette smoking was higher high school students (20.7% males, 6.3% females) than middle school students (7.9% males, 2.8% females). Among high school students, prevalence was higher vocational high school students (31.6% males, 14.8% females) than general high school students (18.2% males, 4.7% females). These results were higher than the HP2020 target of 9.0% for reducing school type differences in smoking [5]. The highest current cigarette smoking prevalence observed among middle and high school students of lower perceived household economic status or lower perceived academic records (sex-adjusted, school grade-adjusted and school type-adjusted) (Table2).

## CONCLUSION

The results from KNHANES and KYRBWS showed that during 2008-2013, the prevalence of current cigarette smoking among male adults did not change significantly from 2008 to 2011 and declined from 2012 to 2013. On contrast, among female adults, the prevalence did not change from 2008 to 2013. Moreover, differences in the prevalence by household income were clearly observed in both males and females. Among adolescents, the prevalence of current cigarette smoking that had remained stable since 2005 showed a decreasing trend after 2011 in both male and female students, although the differences between general and vocational high schools remained high.

The prevalence of current cigarette smoking among male adults was higher than the HP2020 target of 29.0% (difference 13.1% points); among female adults, the prevalence of current cigarette smoking was very close to the HP2020 target of 6.0% [5]. In particular, household income differences exceeded the HP2020 target by approximately 4.0% points in both males and females [5]. Furthermore, regarding the prevalence of current cigarette smoking among adolescents in 2013, the result among male students was higher than the HP2020 target of 12.0% (difference 2.4% points); among female students, the result was lower than the HP2020 target of 6.0% (difference 1.4% points) [5].

To reduce the smoking prevalence, South Korea ratified the Framework Convention on Tobacco Control in 2005 and subsequently amended the National Health Promotion Act to include anti-smoking policies such as expanding smoke-free areas, strengthening cigarette package warnings, and restricting advertisements; furthermore, even stronger policies, such as implementing image-based cigarette package warnings and increasing cigarette prices, are in progress. In addition, continuous monitoring of smoking patterns is planned through KNHANES and KYRBWS.

## Figures and Tables

**Table 1.** Prevalence of current cigarette smoking^1^ among adults in the Korea National Health and Nutrition Examination Survey, 2013

Categories	Total		Male		Female	
Age (yr)						
19–29	701	24.1 (2.2)	312	37.0 (3.1)	389	9.1 (1.7)
30–39	938	30.7 (1.9)	386	54.5 (3.0)	552	6.9 (1.4)
40–49	1040	26.9 (1.6)	446	48.0 (2.4)	594	6.2 (1.1)
50–59	1013	22.0 (1.7)	415	40.8 (2.9)	598	3.7 (0.9)^2^
60–69	847	17.4 (1.5)	378	32.5 (2.8)	469	4.0 (1.0)^2^
≥ 70	799	8.0 (1.0)	317	15.6 (2.1)	482	3.1 (0.8)^2^
Household income (sex-, age-adjusted)						
Low	1298	20.8 (1.5)	550	46.1 (2.3)	748	8.9 (1.3)
Middle-low	1344	17.6 (1.5)	567	42.5 (2.5)	777	6.3 (1.0)
Middle-high	1312	15.4 (1.3)	544	40.2 (2.6)	768	3.9 (0.8)
High	1356	12.6 (1.1)	579	34.3 (2.3)	777	3.5 (0.8)
Education (19–64 yr; sex-, age-adjusted)						
Elementary school and less	464	23.4 (2.8)	122	54.5 (5.2)	342	8.8 (2.2)
Middle school	387	19.5 (2.7)	157	43.8 (4.3)	230	9.8 (2.4)
High school	1696	18.9 (1.4)	739	46.1 (2.2)	957	5.3 (1.0)
College and higher	1587	14.3 (1.1)	731	40.2 (2.3)	856	2.1 (0.5)
Occupation (19–64 yr; sex-, age-adjusted)						
Administrator and professional	637	12.8 (1.6)	309	34.8 (3.5)	328	2.6 (0.8)^2^
Office worker	479	17.1 (2.1)	247	43.6 (3.9)	232	2.8 (1.0)^2^
Service and sales worker	668	25.0 (2.1)	260	51.4 (3.2)	408	10.8 (1.6)
Agriculture, forestry and fishing worker	117	20.0 (4.4)	66	47.1 (7.6)	51	5.0 (4.7)^3^
Technicians and mechanics	481	23.3 (2.4)	413	51.6 (2.8)	68	2.1 (1.4)^3^
Simple laborer	353	22.1 (2.9)	122	49.3 (5.3)	231	8.2 (1.9)

Values are presented as number or % (standard error).

1 Percentage of adults who had smoked at least 5packs of cigarettes (100 cigarettes) during their lifetime and who are currently smokers (aged 19 years and older).

2 Coefficient of variability is 25–50%.

3 Coefficient of variability is >50%.

**Table 2.** Prevalence of current cigarette smoking^1^ among adolescents in the Korea Youth Risk Behavior Web-based Survey, 2013

Categories	Total		Boys		Girls	
School grade						
Grade 7 (middle school)	12199	2.3 (0.2)	6411	3.1 (0.3)	5788	1.4 (0.2)
Grade 8 (middle school)	12113	5.5 (0.3)	6261	7.5 (0.4)	5852	3.3 (0.3)
Grade 9 (middle school)	12218	8.6 (0.4)	6249	13.0 (0.6)	5969	3.8 (0.3)
Grade 10 (high school)	12028	12.1 (0.5)	6098	18.3 (0.7)	5930	5.4 (0.4)
Grade 11 (high school)	11865	14.3 (0.6)	5595	20.8 (0.9)	6270	7.0 (0.5)
Grade 12 (high school)	12012	15.0 (0.7)	6041	22.8 (1.0)	5971	6.4 (0.4)
School type						
Middle school	36530	5.5 (0.2)	18921	7.9 (0.3)	17609	2.8 (0.2)
High school	35905	13.8 (0.5)	17734	20.7 (0.7)	18171	6.3 (0.3)
General high school	29120	11.7 (0.5)	14278	18.2 (0.7)	14842	4.7 (0.3)
Vocational high school	6785	24.3 (1.4)	3456	31.6 (1.5)	3329	14.8 (1.2)
Perceived household economic status (sex-, school grade-, school type-adjusted)						
Low	3388	13.3 (0.6)	1806	20.0 (1.0)	1582	9.5 (0.8)
Middle-low	11806	8.3 (0.3)	5708	13.8 (0.6)	6098	4.8 (0.3)
Middle	34494	6.5 (0.2)	16377	11.4 (0.3)	18117	3.4 (0.2)
Middle-high	17525	6.1 (0.2)	9312	10.8 (0.4)	8213	3.0 (0.2)
High	5222	7.9 (0.4)	3452	12.6 (0.6)	1770	6.0 (0.6)
Perceived academic record (sex-, school grade-, school type-adjusted)						
Low	9185	16.8 (0.5)	4960	25.4 (0.8)	4225	11.5 (0.6)
Middle-low	18107	8.5 (0.3)	8914	14.5 (0.5)	9193	4.7 (0.2)
Middle	20148	5.5 (0.2)	9897	10.1 (0.4)	10251	2.5 (0.2)
Middle-high	17053	4.0 (0.2)	8350	7.5 (0.3)	8703	1.8 (0.1)
High	7942	4.0 (0.2)	4534	6.9 (0.4)	3408	2.4 (0.3)

Values are presented as number or % (standard error).

1 Percentage of students who smoked more than 1 day during the past 30 days (aged 13-18 years).
