# Supplementary material for: Trends in cigarette smoking among adolescents and adults in South Korea
Source: Epidemiol Health. 2014 Oct 28;36:e2014023. doi: 10.4178/epih/e2014023 (PMC4371385; doi:10.4178/epih/e2014023)
Supplement: Supplementary file 1 [file epih-36-e2014023-supplementary.pdf]

# 우리나라 성인 및 청소년의 흡연현황

Trends in cigarette smoking among adolescents and adults in Korea

Sunhye Choi, Suyeon Park, Jihye Lee, Yoonjung Kim, Kyungwon Oh
Division of Health and Nutrition Survey, Korea Centers for Disease Control and Prevention

**서론**

흡연은 암, 심혈관질환 및 호흡기계질환 등 각종 만성질환의 위험요인이며[1], 특히 청소년기 시작된 흡연은 신체적, 정신적 발달에 직접적으로 악영향을 미칠 뿐만 아니라 일탈행위나 불건강행태와 밀접한 관련이 있다[2]. 담배연기에는 4,000 여종이상의 화학물질과 최소 250 여종의 유해물질, 그리고 50 여종이상의 발암물질이 포함되어 있어 간접흡연에 노출된 어린이와 비흡연자는 조기사망과 질병발생 위험이 크다[3,4]. 전 세계적으로 매년 600 만 명이 흡연으로 인해 사망하며, 2030 년에는 한 해 800 만 명 이상이 사망할 것이라 전망하고 있다. 흡연자의 절반은 흡연과 관련된 질병으로, 흡연으로 인해 사망하는 사람 중 10%는 간접흡연에 의한 것으로 보고되고 있다[4].

국제연합(United Nation, UN)은 2025 년까지 비감염성질환에 의한 사망률을 현재 수준대비 25% 감소시키겠다는 목표를 설정하고, 비감염성질환의 예방과 관리에 있어 금연의 중요성을 인식하여 흡연율 감소를 주요 전략에 포함하였다(15 세 이상의 현재흡연율 현재 수준대비 30% 감소목표). 세계보건기구(World Health Organization, WHO)는 오타와헌장 및 방콕헌장 등을 통해 지난 30 여 년간 흡연과 관련된 건강 문제를 이슈화 하고, 건강증진 실천방향을 제시하며 관련 사업을 적극 지원해 왔다. 더불어, 우리나라도 국민건강증진종합계획 2020(Health plan 2020, HP2020)에서 성인 및 청소년의 흡연율을 낮추기 위한 목표를 수립하여 관련 사업을 추진 중에 있으며[5], 국민건강영양조사(Korea National Health and Nutrition Examination Survey)와 청소년건강행태온라인조사(Korea Youth Risk Behavior Web-based Survey)를 통해 성인과 청소년의 흡연현황을 매년 모니터링하고 있다.

이 원고에서는 국민건강영양조사와 청소년건강행태온라인조사의 결과를 중심으로 우리나라 성인과 청소년의 흡연현황을 살펴보고자 한다.

**분석방법**

분석 자료로 국민건강영양조사 제 1 기(1998)부터 제 6 기 1 차년도(2013)와 청소년건강행태온라인조사 2005 년부터 2013 년까지의 원시자료를 이용하였다. 국민건강영양조사는 전국 200 여개 표본조사구 4,000 여 가구를 대상으로 실시하였고, 조사대상자는 해당 조사구의 선정된 20 여 가구, 만 19 세 이상 가구원이다. 흡연영역은 건강설문조사에서 자기기입식으로 조사되었고 본 조사의 참여율은 매년 약 75%였다. 청소년건강행태온라인조사는 전국 800 개 표본학교의 중학교 1 학년부터 고등학교 3 학년까지 약 80,000 명이 익명성자기기입식 온라인조사방법으로 실시하며 응답률은 매년 95%이상이었다. 각 결과는 우리나라 국민을 대표할 수 있도록 가중치를 부여하였으며 국민건강영양조사 추이결과는 2005 년 추계인구로 연령표준화한 값을 제시하였다.

**본론**

성인(만 19 세 이상)의 현재흡연율(연령표준화)은 남자의 경우 2008~2011 년 동안 47% 수준으로 유지되다가 2012 년 43.7%, 2013 년에는 42.1%로 감소추세를 보였다. 여자는 연도별 큰 차이는 없었으나 2013 년 6.2%로 2012 년에 비해 1.7%p 감소하였다(Figure 1). 이는 HP2020 목표인 성인 남자 현재흡연율 29.0%보다 13.1%p 높았고, 여자는 목표 6.0%에 근접한 결과를 보였다[5]. 미국의 경우, 2013 년 성인(18 세 이상)의 현재흡연율은 남자 20.5%, 여자 15.3%로 우리나라 남자는 미국보다 2 배 정도 높았고 여자는 2 배 정도 낮은 경향이었다[6].

청소년(중 1-고 3)의 현재흡연율은 2013 년 남학생 14.4%, 여학생 4.6%로 2012 년에 비해 남학생 1.9%p, 여학생 1.3%p 감소하였다(Figure 1). HP2020 목표인 청소년 현재흡연율에서 남학생은 12.0%보다 2013 년 결과가 더 높았지만, 여학생은 목표 6.0%보다 낮은 수준이었다[5]. 미국의 2013 년 고등학생(9-12 학년)의 현재흡연율은 남학생 16.4%, 여학생 15.0%로 우리나라 고등학교 남학생은 다소 높았고, 여학생은 2.5 배 정도 낮았다[7].

성인(만 19 세 이상)의 현재 비흡연자의 가정실내 간접흡연 노출률(연령표준화)은 2013 년 남자 5.5%, 여자 14.1%로 남자보다 여자가 가정 실내에서 간접흡연에 노출되는 경우가 많았다(Figure 2). 또한 청소년(중 1-고 3)의 주 1 일 이상 가정 내 간접흡연 노출률은 2013 년 남학생 29.5%, 여학생 32.0%로 2008 년 이후 꾸준히 감소하는 경향이지만, 여전히 성인에 비해 2 배 이상 높은 결과를 보였다(Figure 2). HP2020 목표에서는 가정실내 간접흡연 노출률이 남자 1.0%, 여자 5.0%, 청소년 5.0%로 설정되어 있어 현재 수준은 목표보다 높았다[5].

성인(만 19 세 이상)의 현재 비흡연자의 직장실내 간접흡연 노출률(연령표준화)은 2013 년 남자 57.2%, 여자 38.7%로 여전히 비흡연자들의 직장실내 간접흡연 노출은 높은 수준으로 지속되고 있다(Figure 3).

2013 년 성인의 현재흡연율을 연령, 소득수준, 교육수준, 직업별로 분석 한 결과, 연령별로는 남자의 경우 30 대가 54.5%, 여자는 20 대에서 9.1%로 가장 높았다. 소득수준별(성, 연령보정)로는 남녀 모두 소득수준이 낮을수록 현재흡연율이 높게 나타났다. 소득수준 하위계층에서 남자 46.1%, 여자 8.9%이었고, 상위계층에서는 남자 34.3%, 여자 3.5%로 상위계층과 하위계층의 격차는 남자의 경우 11.8%p, 여자는 5.4%p 이었다. 이는 HP2020 목표인 소득 간 차이 남자 8.0%p, 여자 1.5%p 보다 높은 결과이다[5]. 교육수준별(19-64 세, 성, 연령보정)로는 남녀 모두 교육수준이 낮을수록 현재흡연율이 높았고, 대졸이상인 경우 남자 40.2%, 여자 2.1%로 가장 낮았다. 직업군별(19-64 세, 성, 연령보정)로 현재흡연율을 분석한 결과, 남자는 기능원, 장치기계조작 및 조립종사자 51.6%, 서비스 및 판매종사자 51.4%, 단순노무종사자 49.3% 순이고, 여자는 서비스 및 판매종사자 10.8%, 단순노무종사자 8.2%, 농림어업 숙련종사자 5.0% 순이었다(Table1).

청소년(중 1-고 3)의 현재흡연율을 학년, 학교급, 주관적 경제수준 및 학업성적별로 분석한 결과, 학년별로는 고 3 남학생 22.8%, 고 2 여학생 7.0%로 가장 높았다. 학교급별로는 남학생의 경우 중학교 7.9%, 고등학교 20.7%이었고, 일반계고 18.2%에 비해 특성화계고가 31.6%로 약 2 배 정도 현재흡연율이 높았다. 여학생의 경우에는 중학교 2.8%, 고등학교 6.3%이었고 일반계고 4.7%에 비해 특성화계고는 14.8%로 약 3 배 정도 높은 수준이었다. 이는 HP2020 에서 고등학교 계열별 흡연율 차이를 줄이려는 목표 9.0%보다 높았다[5]. 주관적 가정의 경제적 상태(성, 학년, 학교급 보정)와 주관적 학업성적(성, 학년, 학교급 보정)에 따른 결과는 경제적 상태가 낮을수록, 학업성적이 낮을수록 현재흡연율이 높았고, 특히 학업성적의 상위급과 하위급의 차이는 남학생 18.5%p, 여학생 9.1%p 로 크게 나타났다(Table2).

**결론**

국민건강영양조사와 청소년건강행태온라인조사 결과, 성인의 현재흡연율은 여자에서는 연도별 큰 변화를 보이지 않는 반면, 남자의 경우 2008 년부터 답보상태였던 흡연율이 2011 년 이후로 감소경향을 보이나 여전히 40%이상으로 유지되고 있었다. 또한 소득수준에 따른 흡연율의 차이가 남녀 모두에서 뚜렷하게 나타났다. 청소년의 경우에는 2005 년부터 비슷한 수준이던 현재흡연율이 2011 년 이후 남녀학생 모두에서 감소하는 경향이었고 일반계고와 특성화계고 간의 격차는 여전히 컸다.

HP2020 의 목표와 비교 시, 성인의 경우 2013 년 현재흡연율은 목표보다 남자는 13.1%p 높은 결과를 보였고, 여자는 0.2%p 차이로 목표와 비슷한 결과이었다. 특히 소득수준 간 차이는 남녀모두에서 목표보다 약 4%p 정도 높았다[5]. 또한 청소년의 2013 년 현재흡연율은 남학생의 경우 목표보다 2.4%p 높았으나 여학생은 1.4%p 낮은 결과를 보였다. 고등학교 계열별 흡연율 차이도 여전히 2-3 배 높은 수준이었다[5].

우리나라는 흡연율 감소를 위해 2005 년 담배규제기본협약(Framework Convention on Tobacco Control: FCTC)에 비준한 이후 국민건강증진법을 개정하여 금연구역 확대와 담뱃갑 경고문구 강화, 금연광고 제한 등 금연정책을 마련하였고 담뱃갑 경고그림 도입과 담뱃값 인상 등의 보다 강력한 정책도 추진 중에 있다. 또한 국민건강영양조사와 청소년건강행태온라인조사를 통해 흡연현황을 지속적으로 모니터링 할 계획이다.

**참고자료**

1. US Department of Human Health Service. A Report of the Surgeon General, The health consequences of smoking. 2004.

2. US Department of Human Health Service. A Report of the Surgeon General, Preventing tobacco use among young people. 1994.
3. US Department of Human Health Service. A Report of the Surgeon General, The health consequences of involuntary exposure to tobacco smoke. 2006.
4. http://www.who.int/mediacentre/factsheets/fs339/en/imdex.html
5. 보건복지부. 제3차 국민건강증진종합계획 2020. 2011.
6. US Department of Human Health Service, Centers for Disease Control and Prevention, National Center for Health Statistics. Summary Health Statistics for U.S. Adults : National Health Interview Survey, 2013. 2014.
7. Centers for Disease Control and Prevention. Morbidity and Mortality Weekly Report, Youth Report Behavior Surveillance-United States 2013. 2014.
